# Violence against physicians and nurses: a systematic literature review

**DOI:** 10.1007/s10389-021-01689-6

**Published:** 2022-01-22

**Authors:** Sayantan Chakraborty, Saidur Rahman Mashreky, Koustuv Dalal

**Affiliations:** 1 Kolkata Haematology Research Initiatives (KHERI), Kolkata, India; 2 Centre for Injury Prevention and Research Bangladesh (CIPRB), Dhaka, Bangladesh; 3 Division of Public Health Science, School of Health Sciences, Mid Sweden University, Sundsvall, Sweden, and School of Medicine and Health Care, al-Farabi Kazakh National University, Almaty, Kazakhstan

**Keywords:** Violence, Physician, Nurses, Healthcare, Systematic review

## Abstract

**Background:**

Violence against physicians and nurses is a global public health problem. This study explored violence against physicians and nurses using a systematic literature review.

**Methods:**

Pubmed and Scopus were searched using search words ‘violence’ OR ‘aggression’ ‘against’ ‘physicians’ AND ‘nurses’. Articles published between 2010 and 2020 in the English language, excluding review/systemic review articles, were included in the study. We used the Preferred Reporting Items for Systematic Reviews and Meta-Analyses (PRISMA) guidelines for literature search and reporting and assessed the quality of the article based on the JBI checklist for analytical cross sectional studies.

**Results:**

A total of 22 studies were included. The majority of the studies showed that there was a significant violent incident within every setting, often directly involving patients or their relatives. Workers of emergency departments were more likely to be exposed to violence. Verbal abuses were the highest among all settings. Physicians were more likely to face physical violence, while nurses were more prone to sexual harassment. Lack of communication plays a significant role. Fewer reports of violence were noted due to lack of action taken previously.

**Conclusion:**

Adequate policy making and implementation and operational research are required to further mitigate the episodes of violence.

## Background

Violence against physicians and nurses, along with other healthcare personnel, is not a new phenomenon (McKay et al. 2020). Various violent incidents were widely documented worldwide (WHO 2016). An Iranian study indicated that healthcare personnel are 16 times more exposed to violence at their work settings (Najafi et al. 2014). This situation is not only found in middle-income countries, similar incidents happen in high-income countries such as Australia (Hills et al. 2012), the UK (Elston and Gabe 2016), Germany (Vorderwülbecke et al. 2015), and many more. Although every nation has taken a variety of measures to prevent such events, similar incidents continue to occur due to various causes. According to a research paper published from India, some causes of such incidents were low social image of the doctors, the role of media, low health budget constraints and low quality of healthcare. The study also indicated that vulnerability of small and medium private healthcare facilities, lack of faith in the judicial process, low health literacy, huge cost of healthcare and poor service provider–seeker communication trigger the problem (Nagpal 2017). In this Covid-19 context, to prevent workplace violence against healthcare workers, India has passed a new law (Withnall 2020). The patient–physician, along with other healthcare professional relationships, is the keystone of the healthcare delivery system (Agarwal 2017). Therefore, such violent activities are the proxy indicators that a nation has a flawed healthcare delivery system. This review paper provided evidence on the prevalence of different violent activities upon physicians and nurses and their particular characteristics, which will help to devise prevention strategies.

## Methods

The current study has used the Preferred Reporting Items for Systematic Reviews and Meta-Analyses (PRISMA) guidelines for literature search and reporting (Moher et al. 2009). One junior researcher and one senior researcher independently searched scientific literature in November 2020 in two databases viz. PubMed and Scopus using search terms, ‘violence’ OR ‘aggression’ ‘against’ ‘physicians’ AND ‘nurses’. Studies published in English during the past ten years (2010–2020) were screened for inclusion. Eligible studies, both quantitative and qualitative, focused on violence or aggression against physicians or nurses or both were included. Studies related to other healthcare workers along with physicians or nurses or both were also included. Systemic reviews and review studies were excluded.

**Fig. 1:**
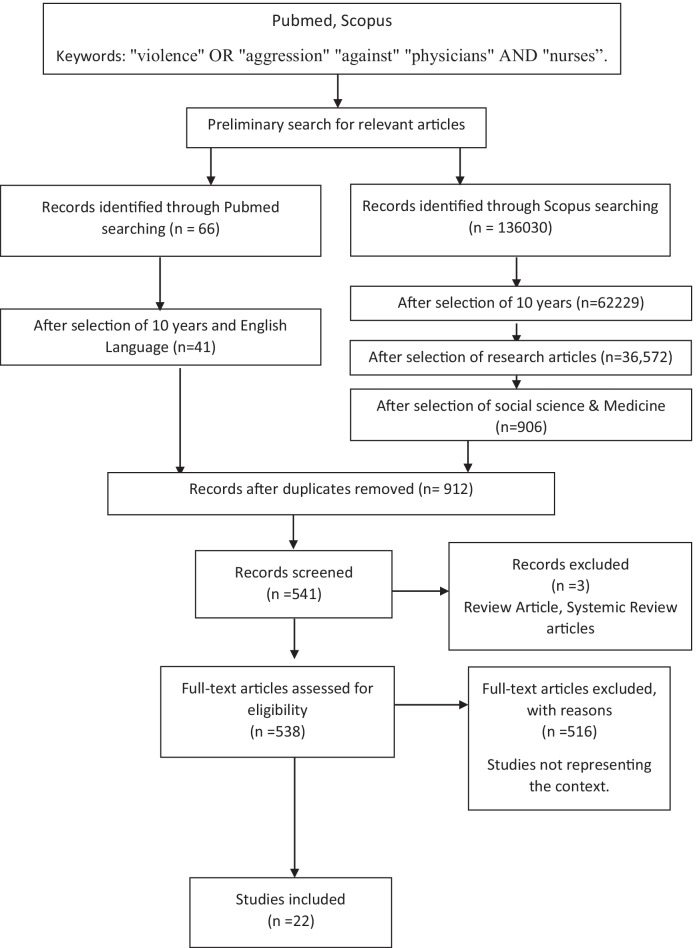
Hierarchicalarticles screening in two search engines and keywords (PRISMA)

One senior researcher rechecked data and consensus by repetitive meetings to resolve all the disagreements and discrepancies. In total, 66 articles were found based on the search strategy in Pubmed and 1360 in Scopus. Among them, after removing duplicates, only 912 articles were selected. Among those articles, 538 were included after excluding non-English, review/systemic review articles, selecting articles published in the past ten years, and selecting fields social science & medicine. Among them, 516 articles were excluded because those articles did not meet the objectives upon which this review study was conducted. Therefore, finally, 22 articles were included for review. The exclusive screening and acceptance process is described in Fig. (1).

The current study focused on and included the author’s name, year of publication, place of study, country, year of study, study type, population, sample size, demographic data, type of violence, prevalence of different types of healthcare workplace violence, vulnerable areas within settings, persons involved in violence and violence initiation, reporting and consequences and suggested strategies.

Three researchers critically assessed the quality of the selected articles based on the JBI checklist for analytical cross sectional studies (Moola et al. 2020). During critical appraisals of the studies, the one junior and one senior researcher independently identified eight broad themes. Another senior researcher rechecked and validated the themes to explore the factors affecting violence against physicians and nurses. All selected articles were presented denoting whether each of them had dealt with those themes.

## Results

Included articles were arranged chronologically according to the year of publication shown in Table 1. The main findings of those articles were written down under the ‘results’ and ‘conclusions’ columns of Table 1.

**Table 1:** Systematic reviewsof selected articlesand authors’ methodology, results and conclusions

Sl No.	• Authors • Year	Methods • Study setting • Study design • Year of study • Sample size • Analysis method used	Results (major findings)	Conclusions
1.	• Kitaneh M, Hamdan M. • 2012	• Five public hospitals located in the five northern districts (Tulkarem, Nablus, Jenin, Qalqilya, Salfit) in the West Bank. • Cross sectional design. • 2011 • 271 (84 licensed physician and 187 nurses) • Pearson’s chi-square analysis was used to test the differences in exposure to physical and non-physical violence according to respondents’ characteristics. Odds ratios and 95% confidence intervals were used to assess potential associations between exposure to violence (yes/no) and the respondents’ characteristics using logistic regression model	• 80.4% reported exposure to violence; 20.8% physical and 59.6% non-physical. • No statistical difference in exposure to violence between physicians and nurses was observed. • Men significantly experienced higher exposure to physical violence in comparison with women. • Logistic regression analysis indicated that less experience (OR 8.03; 95% CI 3.91–16.47) and a lower level of education (OR 3; 95% CI 1.29–6.67) among respondents meant they were more likely to be victims of workplace violence than their counterparts. • The assailants were mostly the patients’ relatives or visitors, followed by the patients themselves, and co-workers. • Consequences of both physical and non-physical violence were considerable. • Only half of victims received any type of treatment. • Non-reporting of violence was a concern, main reasons were lack of incident reporting policy/procedure and management support, previous experience of no action taken and fear of the consequences	• There is a need for intervention to protect health workers and provide safer hospital workplace environments. • The results can inform developing proper policy and safety measures
2.	• Algwaiz WM, Alghanim SA. • 2012	• 2 public hospitals in Riyadh city, Kingdom of Soudi Arabia. • Exploratory cross-sectional survey • 2011 • 383 participants composed of physians and nurses	• 67.4% reported they were victims of violence in the previous 12 months. • Nurses were more likely to be exposed to violent incidents than physicians (*p* < 0.001). • Men, less experienced, and younger respondents were more likely to encounter violent episodes. • Excessive waiting time, shortage of staff and unmet patients’ demands were the most common reasons for violence. • Verbal abuse was the most common type encountered. • The assailants were mostly the patients’ relatives or friends, followed by the patients themselves. • Reasons for not reporting violent events included: feel it is a part of the job, previous experience of no action and fear of consequences	• Physicians and nurses are at high risk of violent incidents. • Health decision makers need to be aware of the potential consequences of such events. • Appropriate preventive measures are needed to make hospitals safer environments
3.	• Zafar W, Siddiqui E et al. • 2013	• Emergency Departments of four of the largest tertiary care hospitals in Karachi, Pakistan. • Cross-sectional survey • 2008 • 266 participants composed of physians and nurses	• Verbal abuse (72.5%), physically attacked (16.5%) - (Among those 29.6% reported that the last incident involved a weapon) and in 64% of cases the attacker was a patient’s relative. • The last attack could have been prevented (86%), and 64% said that no action was taken against the attacker (64%). • Physicians were less likely than nurses to report physical attack (OR 0.46; 95% CI 0.2–1.0), and personnel with greater work experience (OR 4.8; 95% CI 2.0–11.7) and those who said that there were procedures to report WPV in their workplace (OR 3.2; 95% CI 1.6–6.5) were more likely to report verbal abuse. • WPV was associated with mental health effects in the form of bothersome memories, super-alertness and feelings of avoidance and futility	• WPV is an important challenge in the EDs of large hospitals in Karachi. • A majority of respondents feel that WPV is preventable, but only a minority of attackers face consequences
4.	• AbuAlRub RF, Al Khawaldeh AT. • 2014	• Public hospitals in two districs (Ma’an in the south region and Al-Mafraq in the north-east region) of Jordan. • A descriptive exploratory research design - Quantitative study. • 396 nurses and 125 physicians	• 15% of the participants were exposed to physical violence. • The factors - absence of policies, inadequate staffing and lack of communication skills. • Only 16.9% of participants indicated that there were specific policies available for dealing with physical workplace violence. • Strengthening security and providing training were some of the important factors indicated by participants for decreasing violence in the workplace	• Workplace violence is a problem in underserved areas that needs attention from administrators. • Firm policies should be implemented to tackle the problem of workplace violence
5.	• Tucker JD, Cheng Y et al. • 2015	• Seven hospitals in Guangdong Province, China. • Qualitative study using in-depth interviews focused on personal experiences of patient–physician mistrust and trust. • Between June and September 2013. • 160 patients, patient family members, physicians, nurses and hospital administrators at seven hospitals varying in type, geography and stages of achieving goals of health reform. These interviews included purposive selection of individuals who had experienced both trustful and mistrustful patient–physician relationships	• A patient perception of injustice within the medical sphere, related to profit mongering, knowledge imbalances and physician conflicts of interest. • Individual physicians, departments and hospitals were explicitly incentivised to generate revenue without evaluation of caregiving. • Physicians did not receive training in negotiating medical disputes or humanistic principles that underpin caregiving. • Patient–physician mistrust precipitated medical disputes leading to the following outcomes: non-resolution with patient resentment towards physicians; violent resolution such as physical and verbal attacks against physicians; and non-violent resolution such as hospital-mediated dispute resolution. • Policy responses to violence included increased hospital security forces, which inadvertently fuelled mistrust. • Instead of encouraging communication that facilitated resolution, medical disputes sometimes ignited a vicious cycle leading to mob violence. • However, patient-physician interactions at one hospital that has implemented a primary care model embodying health reform goals showed improved patient-physician trust	• Restructuring incentives, reforming medical education and promoting caregiving are pathways towards restoring trust. • In addition to regulatory and legal, responses are urgently needed to restore trust
6.	• Kvas A, Seljak J. • 2015	• Slovenia. • Survey- Quantitative. • 2010–2011 • 692 nurses	• Verbal violence (60.11%) and physical violence (26.01%). • The most frequent perpetrators of verbal violence were patients (listed as a source of violence by 39.3% of the respondents) and peers (39.6%), with the most forceful identified as physicians and patients. • Physical violence against nurses was most often initiated by patients (20.8%) who were also the most forceful source in this category. • Nurse leaders were found to be the most frequent and forceful sources of violence in terms of leadership level. • A positive correlation between external (patient and relatives) and internal (physician and nurse) sources of violence was determined	• Development of action-oriented violence management strategies required
7.	Park M, Cho SH et al. 2015	• University hospital in Seoul, South Korea. • Cross sectional study. • 2013 • 970 female nurses. • Relationships among variables were examined by conducting multiple logistic regression analyses with multilevel modelling	• Verbal abuse (63.8%), threats of violence (41.6%), physical violence (22.3%), sexual harassment (19.7%) and bullying (9.7%). • Physical violence, threats of violence and verbal abuse occurred most frequently in ICUs, whereas sexual harassment and bullying were highest in operating rooms. • The main perpetrators were patients, followed by physicians and patients’ families. • Nurses perceiving greater work demands and less trust and justice were more likely to have been exposed to violence	• Reduction of violence will contribute to creating a better nursing work environment
8.	• Hamdan M, Abu Hamra A. • 2015	• Main 14 hospitals having Emergency Departments (8 from the West Bank (WB) and 6 from the Gaza Strip (GS). • Cross sectional study. • 2013 • A total of 444 participants (response rate 74.5%): 161 (32.0%) nurses, 142 (32%) physicians and 141 (31.7%) administrative personnel. • Multivariate regression analysis was performed	• 76.1% experienced a type of WPV: physical (35.6%) and 71.2% to non-physical assaults (69.8% verbal abuses, 48.4% threats and 8.6% sexual harassments). • Perpetrators of physical and non-physical violence were mainly patients’ families/visitors (85.4% and 79.5%, respectively). • Waiting time, lack of prevention measures and unmet expectations of patients and their families are the main reasons for WPV. • The multivariate regression analysis showed that younger personnel (OR = 2.29 CI 95% 1.309–4.036), clinicians (nurses and physicians) (OR = 1.65 CI 95% 0.979–2.797) compared with administrative and less experienced ED personnel (OR = 2.39 CI 95% 1.141–5.006) are significantly at higher risk of exposure to WPV (P < 0.05). • Low level (40%) of violence reporting is evident, largely attributed to not enough actions being taken and fear of consequences. • Violence has been shown to have considerable consequences for workers’ well-being, patient care and job retention	• Internal-system-related factors are the most amenable to change. • Attention should be given to strengthening violence prevention policy and measures and improving incident-reporting system
9.	• Pompeii LA, Schoenfisch AL et al. • 2015	• Two large hospital systems in Texas (TX) and North Carolina (NC); each system included one large medical centre hospital and two community hospitals- Total 6 Hospitals. • Cross sectional survey. • 11,000 hospital workers	• 39% experienced violence; 2098 of 5385 workers experienced 1180 physical assaults, 2260 physical threats and 5576 incidents of verbal abuse. • Direct care providers were at significant risk. • Perpetrator circumstances attributed to violent events included altered mental status, behavioural issues, pain/medication withdrawal and dissatisfaction with care. • Fear for safety was common among worker victims (38%). Only 19% of events were reported into official reporting systems	• This pervasive occupational safety issue is of great concern and likely extends to patients for whom these workers care for
10.	• Abed M, Morris E et al. • 2016	• Eight government-owned primary care clinics in Barbados. • Cross sectional study. • 2014 • 102 participants	• 63% of nurses and physicians reported at least one episode of violence in the past year. • Verbal abuse (60%) and bullying (19%), sexual harassment (7%), physical violence (3%) and racial harassment (3%). • Patients emerged as the main perpetrators of violence (64%). • Significant associations between gender and workplace violence. Women and nurses were more predisposed to experience violent incidents than men and physicians	• Female gender being a significant predictor of abuse. • Adequate documentation and implementing clear policies and violence prevention programmes in health institutions are crucial steps towards addressing this issue
11.	• Ferri P, Silvestri M et al. • 2016	• A general hospital in northern Italy. • Cross sectional study. • 2015 • 419 health professionels who worked in 15 departments. • Logistic regression was performed	• 45% of professionals reported WPV. Nurses (67%), nursing assistants (18%) and physicians (12%). • The first two categories were correlated, in a statistically significant way, with the risk of WPV (*p* = 0.005, *p* = 0.004, multiple logistic regression). • The violent incidents more frequently occurred in the psychiatry department (86%), emergency department (71%) and in geriatric wards (57%). • The assailants more frequently were men whereas assaulted professionals more often were women. Men committed physical violence more frequently than women, in a statistically significant way (*p* = 0.034, chi-squared test). • Verbal violence (51%) was often committed by people in a lucid and normal state of consciousness; physical violence (49%) was most often perpetrated by assailants affected by dementia, mental retardation, drug and substance abuse, or other psychiatric disorders. • The variables positively related to • WPV were ‘calling for help during the attack’ and ‘physical injuries suffered in violent attack’ (*p* = 0.02, *p* = 0.03, multiple logistic regression)	• This study suggests that violence is a significant phenomenon and that all health workers, especially nurses, are at risk of suffering aggressive assaults. • Prevention programmes tailored to the different care needs are necessary to promote professional awareness for violence risk
12.	• Bilici R, Sercan M et al. • 2016	• Locked Psychiatric Clinics of Turkey. • Cross sectional survey. • 137 participants (62 nueses, 50 physicians and 25 health officers). • Chi squre test was performed	• 87.6% of staff members viewed security measures insufficient. • Preventive actions should be taken to reduce the risk of exposure to violence against the staff members working at the locked psychiatric clinics	• Preventive actions should be taken to reduce the risk of exposure to violence against the staff members working at the locked psychiatric clinics
13.	• Sun T, Gao L. • 2017	• 30 provinces of China. • A cross-sectional online survey study. • 2016 • 2617 doctors. • Pearson correlation & multiple hierarchical linear regression was done	• Verbal abuse (76.2%), made difficulties (58.3%), smear reputation (40.8%), mobbing behaviour (40.2%), intimidation behaviour (27.6%), physical violence (24.1%) and sexual harassment (7.8%). • WPV significantly affected the psychological stress, sleep quality and self-reported health of doctors. • Psychological stress partially mediated the relationship between work-related violence and health damage	• A safer work environment for Chinese healthcare workers needs to be provided to minimise health threats, which is a top priority for both government and society
14.	• Shafran-Tikva S, Chinitz D et al. • 2017	• Hadassah University Medical Center, Kiryat, Hadassah, Jerusalem, Israel. • Mixed method study (Qualitative- FGD, IDI) • 2010 • Participants- For quantitative 676 (230 physicians, 446 nurses), Qualitative – Focus group discussion 20 (Head nurses-5, Physician-5, Staff nurses-5, Sequrity personel-5), In depth interview-18, Open ended questions- 676	• Important factors - staff behaviour (39%), patient behaviour (26%), hospital setting (17%), professional roles and waiting times (10%). • Patients and staff reported similar perceptions and emotions regarding the episodes of violence. 35% of the staff responded that paients contributed to the creation of the most severe violent episode, 48% stated that staff behaviour contributed to violent episodes. • According to physicians and nurses- 50% of violence was related to patient dissatisfaction with the quality of service, the degree of staff professionalism, or an unacceptable comment of a staff member. • Other reasons- lack of understanding of the hospital system on the part of patients, together with poor communication between patients and providers and expectations gaps	• Staff and patients share conditions of overload, pressure, fatigue, and frustration. Lack of coping tools to prevent violence. • Self-conscious awareness regarding potential interacting factors required to coping with hospital violence
15.	• Rafeea F, Al Ansari A et al. • 2017	• Emergency Department of the Defence Force (BDF) Hospital, Bahrain. • Cross sectional exploratory study. • 100 workers of Emergency departments	• Participants experienced verbal abuse (78%), physical abuse (11%) and then sexual abuse (3%). • Violence against ED workers occurred during night shifts (53%), while physical abuse was reported to occur during all the shifts. • 40% of the staff in the ED of the hospital were not aware of the policies against workplace violence, and 26% of the staff considered leaving their jobs at the hospital	• The results clearly demonstrate the importance of addressing the issue of workplace violence in EDs in Bahrain and can be used to demonstrate the strong need for interventions
16.	• Wong AHW, Combellick J. • 2017	• Urban, tertiary care public hospital in New York City, New York. • Focus group discussion and individual interviews using a phenomenological approach. • 31 participants (9 hospital police officers, 10 nurses, 6 patient care technicians and 6 emergency medicine resident physicians)	• Emergency Department’s healthcare workers provide high-quality care to a marginalised patient population that concurrently poses safety threats, creating a patient care paradox. • Teamwork is critical to safely managing this population, but hierarchy and professional silos hinder coordinated care between healthcare professionals. • Environmental challenges and systems issues both in and outside the ED exacerbate threats to safety	• The experience of ED staff members while caring for agitated patients is complex and multidimensional. • Study identified issues that coalesced into four tiers of healthcare delivery at the individual, team, environment and system levels
17.	• Alsaleem SA, Alsabaani A et al. • 2018	• Two government hospitals and ten primary healthcare centres of Abha city, Saudi Arabia. • Cross sectional study. • 738 workers (151 were selected from ten PHCs and 587 from two government hospitals) • A multiple logistic regression modelling was done to identify the predictors of violence against healthcare workers	• 64.9% participants were females and 69.4% were Saudis. • 57.5% participants had experienced some workplace violence at least once. • Verbal assaults and slaps were the most common form of workplace-related violence (58%)	• The reasons need to be explored in order to set and develop policies, regulations and interventions to prevent violence against workers
18.	• Wang N, Wu D et al. • 2018	• Eight county hospitals in four counties, two each from developed counties, Shengzhou and Ninghai, and developing counties, Jiangshan and Kaihua, Zhejiang province, eastern China. • Cross sectional survey. • 2016–2017 • 1388 Health worker. • Odds ratio and Pearson’s correlation was performed	• Physical attacks (7.8%), physical threats (21.2%), 51.6% experienced (medical disturbance created by gangs using extreme means to obtain compensation from a hospital) at least once in the past year. • Physical attacks were significantly more in physicians (10.9%) than in nurses (5.9%). Physicians were threatened at 27.1%, followed by nurses at 20.2%. • Compared with general medicine, health workers working in emergency were significantly more likely to suffer physical attacks ( OR = 2.7, 95% CI = [1.4, 5.2], *p* < .01) and WPV (physical attacks or threats; OR = 2.5, 95% CI = [1.6, 4.1], *p* < .001). • Being encouraged to tolerate WPV was correlated with physical attacks (OR = 6.1, 95% CI = [3.5, 10.4], *p* < .001) and WPV (OR = 6.7, 95% CI = [4.6, 9.8], *p* < .001)	• This highlights the need for a focused systematic prevention concerning health workers’ safety and governmental regulations and the need for hospitals to encourage their employees to report the WPV
19.	• Honarvar B, Ghazanfari N et al. • 2019	• Three main university-affiliated public hospitals in Shiraz, southern Iran—Faghihi, Nemazee and Rajaei Hospitals. • Quantitative study. • 2017–2018 • 405 nurses	• 89.6% nurses had experienced at least one kind of violence; 68.4% suffered from more than one type of violence. • Verbal abuse (83.9%), verbal threats (27.6%), physical violence (21.4%), sexual abuse (10.8%) and ethnical harassment (6.1%). • Patients’ companions, patients and physicians were reported as the sources of violence in 70.6%, 43.1% and 4.1% of cases, respectively. • Nurses with non-official employment status and non-Farsi ethnicity, having a disease, with non-evening shift work, and those with short or long employment period were more affected. • Unrealistic expectations by patients’ companions and long working hours were the most common attributing factors	• Violence against nurses, as a strenuous and health-threatening crisis, should be investigated to shot out the problem
20.	• Sachdeva S, Jamshed N et al. • 2019	• Tertiary care hospital in Delhi, India. • Cross sectional survey. • 2017 • 235 (123 doctors, 112 nurses) • Chi-square and Fisher’s exact tests were used for bivariate analysis while logistic regression analysis was to analyse the impact of violence with participant characteristics	• Verbal abuse (67%), physical assault (17%) was reported by 17% (40/235), while confrontation (11%). • Family members were the main perpetrator for VA (75%) and PA (35%). • Regarding reporting, the violent incidences were mostly reported to ED security and ED faculty. • Individuals who are younger, less experience, and male gender were more exposed to abuse both VA and PA at *p* < 0.05. • Nurses and junior residents reported more abuse than senior residents (*p* < 0.05). • Majority of the participants had reported lack of job satisfaction due to verbal abuse (*p* = 0.01)	• Work place violence is common in Emergency Departments of the current setting. • It results in significant physiological and psychological effects on healthcare providers
21.	• Demirci Ş, Uğurluoğlu Ö. • 2020	• A public hospital in Ankara, Turkey • Cross sectional survey • 347 (104 physicians, 93 nurses and 150 other medical staff) • A logistic regression analysis was performed to determine the effects of sociodemographic features on violence	• Physicians (96.2%), nurses (95.7%) and other medical personnel (80.7%) reported verbal violence at least once in their professional life. • Physicians were identified as a source of violence among other healthcare professionals. • Nurses are exposed to sexual violence more than other medical personnel are (OR = 3.11, 95% CI [1.29, 7.49]). • Nurses were more exposed to verbal (OR = 5.08, 95% CI [1.54, 16.75]) and physical (OR = 3.68, 95% CI [1.15, 11.80]) violence compared with other medical personnel	• This study shows that a great majority of healthcare professionals are subjected to violence ranging from verbal violence in particular to physical and sexual violence
22.	• Davey K, Ravishankar V et al. • 2020	• 7 hospital Emergency Department’s across India. • Qualitative- Semi-structured interviews and hybrid thematic analysis approach was used to determine dominant themes. • 63 participants (11 attending physicians, 36 resident physicians, 10 nurses, and 5 paramedics, 1 interview participant did not give their job title)	• Most events involved verbal abuse, although a significant percentage of responses described some kind of physical violence. • ED factors such as busy times with high patient volumes or periods of waiting are associated with increased violence, as well as incidents with unanticipated outcomes such as patients with severe illness or death. • Decreased levels of health literacy among patients often contribute as well as the financial stressors of paying for medical care. • Providers reported negative consequences of workplace violence on quality of care for patients and their own motivation to work in the ED. • Communication strategies were frequently proposed as interventions to mitigate violence in the future, including both provider communication as well as public awareness campaigns	• Alarming levels of verbal and physical abuse and their impact on patient care are described. • Indian ED providers that differ from those in more developed settings, including financial stressors, inadequate enforcement of rules governing behaviour in the hospital, and an overwhelming frequency of violence emanating from patient family members and attendants rather than the patients themselves

Among those selected studies, 18 studies were cross-sectional, three were qualitative and one study was from the mixed-method domain. Among 22 studies, 18 studies were conducted with physicians and nurses, three studies with nurses and only one study with physicians. Male participants outnumber female participants, if all studies are take into consideration. All studies support that violence was a common phenomenon in all the settings. The majority of the respondents indicated that they were exposed to at least one type of violence in their hospitals during the previous 12 months. There were mainly two types of violence that occurred in the hospitals, viz. non-physical and physical. Non-physical violence mainly includes verbal abuse and threats and physical violence includes direct physical attacks upon health workers. Men were more exposed to physical violence than women healthcare staff. From the angle of sexual harassment, women were more exposed. One study revealed that verbal abuses were more common against married, unhealthy and older nurses (Honarvar et al. 2019). In every setting, there were fewer reports against violence than the prevalence of violence. Tjhe main findings of the included studies were arranged in a tabular fashion according to the date of publication.

This study has identified eight broad areas where the articles are reporting or focusing. The areas are Types and prevalence of violence, Vulnerable groups, Place of violence in health facilities, Source of violence initiation, Causes of violence, Reporting of violence, Consequences, Mitigation strategies.

Table 2 presents the assessment of the quality of the selected articles based on the JBI checklist for cross sectional studies. Our assessment shows that not a sigle study had clearly identified the confounding factors and therefore did not mention strategies to deal with confounding factors. For seven studies, we could not assure that the outcomes were measured in a valid and reliable way.

**Table 2:** Critical appraisal of the selected 22 articles based on JBI checklist

Sl. No.	Inluded artiles	Crit-1	Crit-2	Crit-3	Crit-4	Crit-5	Crit-6	Crit-7	Crit-8
		Were the criteria for inclusion in the sample clearly defined?	Were the study subjects and the setting described in detail?	Was the exposure measured in a valid and reliable way?	Were objective, standard criteria used for measurement of the condition?	Were confounding factors identified?	Were strategies to deal with confounding factors stated?	Were the outcomes measured in a valid and reliable way?	Was appropriate statistical analysis used?
1.	Kitaneh M, Hamdan M.	1	1	1	1	0	X	1	1
2.	Algwaiz WM, Alghanim SA.	1	1	1	1	0	X	1	1
3.	Zafar W, Siddiqui E et al.	1	1	1	1	0	X	1	1
4.	AbuAlRub RF, Al Khawaldeh AT.	1	1	1	99	0	X	1	99
5.	Tucker JD, Cheng Y et al.	1	1	1	1	0	X	99	1
6.	Kvas A, Seljak J.	1	1	1	1	0	X	1	1
7.	Park M, Cho SH et al.	1	1	1	1	0	X	99	99
8.	Hamdan M, Abu Hamra A.	1	1	1	1	0	X	1	1
9.	Pompeii LA, Schoenfisch AL et al.	1	1	99	99	0	X	99	1
10.	Abed M, Morris E et al.	1	1	1	1	0	X	99	1
11.	Ferri P, Silvestri M et al.	1	1	1	1	0	X	1	1
12.	Bilici R, Sercan M et al.	1	1	1	1	0	X	99	1
13.	Sun T, Gao L et al.	1	1	1	1	0	X	99	1
14.	Shafran-Tikva S, Chinitz D et al.	1	1	1	1	0	X	1	1
15.	Rafeea F, Al Ansari A et al.	1	1	99	1	0	X	1	1
16.	Wong AHW, Combellick J et al.	1	1	1	1	0	X	1	1
17.	Alsaleem SA, Alsabaani A et al.	1	1	1	1	0	X	99	1
18.	Wang N, Wu D et al.	1	1	1	1	0	X	1	1
19.	Honarvar B, Ghazanfari N et al.	1	1	99	1	0	X	1	1
20.	Sachdeva S, Jamshed N et al.	99	1	1	1	0	X	1	1
21.	Demirci Ş, Uğurluoğlu Ö.	1	1	99	1	0	X	1	1
22.	Davey K, Ravishankar V et al.	1	1	1	1	0	X	1	1

Yes = 1, No = 0, Unclear = 99 and Not applicable = X

Table 3 presents the conformity of the selected articles with contracted themes. The majority of the selected articles had the following themes, i.e. ‘type and prevalence of violence (17 articles)’, ‘vulnerable groups (13 articles)’, ‘source of violence initiation (12 articles)’ and ‘causes of violence (10 articles)’. Less conformed themes were ‘reporting of violence (8 articles)’, ‘consequences (6 articles)’, ‘place of violence in health facilities (4 articles) and ‘mitigation strategies’ (3 articles).

**Table 3:** Articles’ concepts and themes

Themes Studies (author names)	Types and prevalence of violence	Vulnerable groups	Place of violence in health facilities	Source of violence initiation	Causes of violence	Reporting of violence	Consequences	Mitigation strategies
Kitaneh M, Hamdan M	✓	✓		✓		✓		
Algwaiz WM, Alghanim SA	✓	✓		✓	✓	✓		
Zafar W, Siddiqui E et al.	✓	✓		✓		✓		
AbuAlRub RF, Al Khawaldeh AT	✓				✓	✓		
Tucker JD, Cheng Y et al.					✓		✓	✓
Kvas A, Seljak J	✓			✓				
Park M, Cho SH et al	✓	✓		✓				
Hamdan M, Abu Hamra A	✓	✓		✓	✓	✓	✓	
Pompeii LA, Schoenfisch AL et al.	✓				✓	✓		
Abed M, Morris E et al.	✓	✓		✓				
Ferri P, Silvestri M et al.	✓	✓	✓	✓				
Bilici R, Sercan M et al.					✓			✓
Sun T, Gao L	✓						✓	
Shafran-Tikva S, Chinitz D et al.				✓	✓			
Rafeea F, Al Ansari A et al.	✓	✓				✓	✓	
Wong AHW, Combellick J			✓		✓			
Alsaleem SA, Alsabaani A et al.	✓	✓	✓					
Wang N, Wu D et al.	✓	✓						
Honarvar B, Ghazanfari N et al.	✓	✓		✓	✓			
Sachdeva S, Jamshed N et al.	✓	✓		✓		✓	✓	
Demirci Ş, Uğurluoğlu Ö	✓	✓	✓	✓				
Davey K, Ravishankar V et al.					✓		✓	✓

## Discussion

There was a significant violent incident within every setting. Patients and patient relatives were directly involved. Workers of emergency departments were more likely to be exposed to violence. Verbal abuses were the highest in all settings. Physicians were more likely to face physical violence, while nurses were more prone to sexual harassment. Lack of communication plays a significant role. Fewer reports of violence were noted due to lack of action taken previously.

### Themes

The current study identified eight significant themes based on the review articles. Themes were arranged systematically as type and prevalence of violence, vulnerable group, area of violence, source of violence, causes of violence, reporting of violence, consequences of violence and mitigation strategies.

### Type and prevalence of violence

The majority of the participants in different studies agreed that they faced at least one kind of violent attack in the past 12 months. Significant violence were non-physical assaults, followed by physical assaults. Studies showed that according to prevalence, the most common type of violence was verbal abuse, as indicated in various study settings. Other types of violence were verbal threats, physical assaults, sexual abuse, ethnical harassment, reputation smearing, mobbing behaviour, bullying, intimidation behaviour and racial harassment. Some studies showed that sexual violence was higher than physical violence (Abed et al. 2016), and some showed the opposite (Sun et al. 2017). According to one study, patient’s and staff’s perceptions and emotions regarding violence episodes were similar (Shafran-Tikva et al. 2017). A systemic review and meta-analysis conducted by Liu et al. (2019) showed that 61.9% reported exposure to any form of work place violence such as exposure to non-physical violence was 42.5% and 24.4% experienced physical violence during the previous 12 months. The most common form of non-physical violence was verbal abuse, followed by threats and sexual harassment (Liu et al. 2019). A systemic review and meta-analysis showed similar results that the prevalence of verbal abuse against the emergency department nurses was 89.7% and physical violence was 21.0% (Azami et al. 2018).

### Vulnerable groups

Studies showed that direct care providers were at significant risk (Pompeii et al. 2015). The majority of the studies showed that nurses and women were more likely to be exposed to violence than physicians and men (Algwaiz and Alghanim 2012; Park et al. 2015; Abed et al. 2016; Ferri et al. 2016; Alsaleem et al. 2018; Honarvar et al. 2019; Demirci and Uğurluoğlu 2020). However, another study observed no significant difference between physicians and nurses regarding the episodes of violence (Kitaneh and Hamdan 2012). Physicians and men were more likely to be exposed to physical violence (Kitaneh and Hamdan 2012; Abed et al. 2016;Wang et al. 2018). Nurses and women were more likely to be exposed to sexual harassments (Demirci and Uğurluoğlu 2020). Though one study said physicians were less likely to be exposed to physical attack than nurses (Zafar et al. 2013). Younger and less experienced groups were more exposed to violence than seniors (Kitaneh and Hamdan 2012; Hamdan and Abu Hamra 2015; Sachdeva et al. 2019). However, one study indicated healthcare personnel with more significant work experience were more likely to report verbal abuses (Zafar et al. 2013). Commonly, emergency workers faced violence during the night shift, but workers faced violence in all shifts in other departments (Rafeea et al. 2017).

Vulnerable groups were exposed to violence more due to having less experience regarding better handling the situation, while seniors said their political influence and respect from patients and relatives because of their long years of experience play a significant role (Anand et al. 2016; Kumar et al. 2016; Phillips 2016).

### Place of violence in health facilities

Studies showed that verbal abuse, threats of violence and physical violence most frequently occurred in ICUs, whereas reported highest cases of sexual harassment and bullying were in operating rooms (Demirci and Uğurluoğlu 2020). Those who worked in psychiatry departments, emergency departments, i.e. those who provide a high quality of medical care, and geriatric departments were more likely to be exposed to physical violence (Ferri et al. 2016; Wong et al. 2017; Alsaleem et al. 2018). A systemic review and meta-analysis showed that psychiatry and emergency departments were the most common workplace violence areas (Liu et al. 2019). Another review study supports that emergency departments had a possible chance of becoming a place of violence because of various environmental risk factors of violence such as poor staffing, lack of privacy, overcrowding and availability of nonsecured equipment types that can be used as a weapon (Stowell et al. 2016).

The number of beds in the department in which the critical patients are admitted is usually fewer. Explanation of the patient’s particular situation is not always possible by doctors and nurses in those departments due to time constraints and many more related factors. Owing to this, there is more misunderstanding between patient parties and service providers in those departments. The majority of the outcome of those departments’ cases were low due to the patient’s critical condition leading to more violence in those departments.

### Source of violence initiation

The majority of the studies showed that patients, patient relatives, peers and family members were the main perpetrators of violence (Algwaiz and Alghanim 2012; Kitaneh and Hamdan 2012; Hamdan and Abu Hamra 2015; Kvas and Seljak 2015; Park et al. 2015; Abed et al. 2016; Sachdeva et al. 2019). One of the studies revealed the main perpetrators of violence against nurses were the patient and higher authority and a positive correlation between external, i.e. patient and relatives, and internal, i.e. physician and nurse sources of violence (Kvas and Seljak 2015). Along with the above, physicians also identified as a source of violence among other healthcare providers (Honarvar et al. 2019; Demirci and Uğurluoğlu 2020). Staff behaviours also contributed as a source of violence (Shafran-Tikva et al. 2017). Violence was associated with mental health effects as bothersome memories, super-alertness, feelings of avoidance and futility, etc. Patients affected by dementia, mental retardation, drug and substance abuse, or other psychiatric disorders were more involved with violence (Zafar et al. 2013; Ferri et al. 2016).

### Causes of violence

Most of the studies showed that excessive waiting time was the most common attributor of violence against healthcare staff (Algwaiz and Alghanim 2012; Hamdan and Abu Hamra 2015; Shafran-Tikva et al. 2017; Davey et al. 2020). Other factors were unrealistic expectations from patient parties (Hamdan and Abu Hamra 2015; Shafran-Tikva et al. 2017; Honarvar et al. 2019; Davey et al. 2020), poor understanding by the patients and their families of the healthcare system, poor communication between patients and service-providers (Hamdan and Abu Hamra 2015; Tucker et al. 2015; Shafran-Tikva et al. 2017), lack of communication skills among healthcare providers (AbuAlRub and Al Khawaldeh 2014; Hamdan and Abu Hamra 201515; Tucker et al. 2015), behaviour (Pompeii et al. 2015; Shafran-Tikva et al. 2017), condition of a hospital setting (Shafran-Tikva et al. 2017; Wong et al. 2017), staff professionalism, including their unacceptable comments and clients dissatisfaction about the quality of services (Pompeii et al. 2015; Shafran-Tikva et al. 2017), absence of policies, inadequate staff (Algwaiz and Alghanim 2012; Bilici et al. 2016) and long working hours (Honarvar et al. 2019). Another systemic review found a similar result that major risk factors of violence were long waiting times, the discrepancy between patients’ expectations and services, substance abuse by the patient and their psychiatric conditions (Raveel and Schoenmakers 2019). Another review study indicated that patients’ psychological health, including anxiety, acute stress reaction, alcohol and drug intoxication and dementia, were predictors of physical violence against healthcare workers perpetrated by patients (D’Ettorre et al. 2018).

### Reporting of violence

Violence was most often reported by security personnel and emergency department staff (Sachdeva et al. 2019). Lack of violence reporting was present in every setting. One study showed that only 19% of violent incidents were reported (Pompeii et al. 2015). The review revealed that physicians and nurses were not interested in reporting violence due to various reasons such as the previous experience of no action taken and fear of the consequences and lack of management support (Algwaiz and Alghanim 2012; Zafar et al. 2013; AbuAlRub and Al Khawaldeh 2014; Hamdan and Abu Hamra 2015). They were not aware of reporting policy & procedures (Kitaneh and Hamdan 2012; Rafeea et al. 2017).

### Consequences

Owing to consequences of violence, healthcare providers reported lack of job satisfaction. According to one study, 26% of the staff considered leaving their job at the hospital (Rafeea et al. 2017; Sachdeva et al. 2019). Workplace violence significantly affected the psychological stress, sleep quality and health worker–patient relationship significantly, ultimately affecting the quality of patient care services (Hamdan and Abu Hamra 2015; Tucker et al. 2015; Sun et al. 2017; Davey et al. 2020). Similar findings were shown in a systemic review and meta-analysis conducted by Binmadi and Alblowi (2019) that the impact of violence on workers manifested as impaired quality of work, psychological problems, and, although rare, quitting the job. Another review study conducted by Baydin and Erenler (2014) showed a similar result that workplace violence victims’ most common psychological effects were reduced job satisfaction and fear.

### Mitigation strategies

Prevention strategies should be taken to reduce the violent attacks against physicians and nurses and other healthcare providers, mainly those who work in emergency departments (Bilici et al. 2016). Strengthening security personnel and training to mitigate workplace violence among providers is required (Tucker et al. 2015). Communication strategies should be devised as well as public campaigns required to nullify the communication gaps (Davey et al. 2020). Similar mitigation strategies were expressed by the findings of a systemic review and meta-analysis that governments, policymakers and health institutions need to take actions to address work place violence towards healthcare professionals globally (Liu et al. 2019). However, another systemic review and meta-analysis showed no hard evidence on the effectiveness of that (Raveel and Schoenmakers 2019). A qualitative meta-analysis conducted by Ashton et al. (2018) recommended similar findings to reduce workplace violence staff training in understanding violence and aggression and clinical supervision needed. Along with the above, another review study conducted by Gillespie et al. (2010) suggested other protective strategies to combat the negative consequences of violence such as carrying a telephone, practising self-defence, instructing perpetrators to stop being violent, self and social support, and limiting interactions with potential or known perpetrators of violence.

### Limitations

There were many publications related to our topic on other sites. However, we selected only Pubmed and Scopus indexed journals to conduct this review because Pubmed is a free accessed website and Scopus for its popularity in medicine and social science. Another limitation of our review study is that we did not include those studies conducted on health workers other than doctors and nurses. Because doctors and nurses are directly involved in service delivery and are primary victims of healthcare workplace violence, most of the studies concerned about such a topic had taken samples from the doctor and nurse communities. However, to explain the ground reality and context, much more inclusion of other healthcare providers’ studies are necessary. The study findings address patient and family as sources of violence, but does not address co-worker or non-client violence (Phillips 2016). Also, types of violence varies across countries. Different countries have different cultures and contexts of violence against physicians and nurses. Therefore, more context-based exploration of the causes of violence against physicians and nurses are warranted, especially in the low and middle income countries.

### Proposed checklist for scientific research on violence against physicians and nurses (VPNCheck)

From our search strategy in the two most widely used databases and most appropriate search words, based on study objectives, we have initially critically assessed the selected articles. During the review, we explored specific gaps among included articles. We propose a checklist (Table 4) for scientific research on violence against physicians and nurses (VPNCheck).

**Table 4:** Proposed checklist for scientific research on violence against physicians and nurses (VPNCheck)

SL No.	Heading	Points should be covered	Remarks
1.	Introduction	Must clearly present the background of the study with rationale for the study	
2.	Objective	Clearly state the objective of the study	
3.	Methodology	Study design should be properly described. Interviews should be taken away from the patient’s bed preferably in separate room to explore real picture better. Education/Academic qualification should be explored side by side experiences/number of years practices because both the issues are not the same. Intern and trainee doctors should be included into the study because they faced the patient much more than seniors in most of the tertiary care centres Male nurses should be included though they were not the majority and females to explore the influence of gender variable better. In the case of telephone interviews/ self-administered questionnaire, data quality should be checked to address contamination or regression of mean bias. Questionnaire selection should be comprehensive, covering all aspects of workplace violence related to the selected population. Qualitative studies should not be exclusively based on interview guide. It should be flexible after contextualization based on ground reality. Academic qualification/degree should be included and experience of work to know the relation of educational qualification with the issue	Include: witness-based study. Part-time staff along with full-time staff
4.	Result	Issue related to fewer reports of violence came up repeatedly. One reason to be less likely to report was due to ignorance from higher authority, a nurse or doctor representative. However, why the issue of ignorance repeatedly came up, which leads to non-reporting by victims, was not explored properly among selected articles. This issue should be focused on more.	
5.	Discussion	Negative findings should be discussed along with common positive findings to explore the situation as a whole. Methodological limitations and recommendations for future studies based on own experience should be included	
6.	Conclusion	Strength and weakness of the current study should be written down along with limitations. Conclusion should be exclusively based on study findings	

## Conclusion

Reduction of violence provides a better working environment for healthcare workers leading to a better healthcare delivery. Internal system-related factors should be considered first. Emergency departments prone to violent attacks should intervene first. Context-based research guiding strategies and policies are needed to reduce workplace violence against physicians and nurses.

## Data Availability

For literature review all selected articles are availabel in open system.

## Abbreviations

UK: United Kingdom of Great Britain and Northern Ireland.
ICU: Intensive Care Unit.

## Code availability (software application or custom code)

N/A
